# Supplementary material for: Resolving the full spectrum of human genome variation using Linked-Reads
Source: Genome Res. 2019 Apr;29(4):635–45. doi: 10.1101/gr.234443.118 (PMC6442396; doi:10.1101/gr.234443.118)
Supplement: Supplemental Material [file supp_gr.234443.118_Supplemental_longranger-2.2.zip › longranger-2.2.tar.bz2!/lr2.2-public/lib/go/src/code.google.com/vitess/web/vtctld2/src/app/dashboard/tablet.component.html]

### Loading...

# {{tablet.label}}

Delete
Ping
Refresh State
SetReadOnly
SetReadWrite
StartSlave
StopSlave
RunHealthCheck
IgnoreHealthError
ReparentTablet

Go to Tablet
